# Neuropharmacological effects of standardized aqueous stem bark extract of *Parkia biglobossa* in Wistar rats

**Published:** 2014

**Authors:** Tijani Adeniyi Yahaya, Samuel Ehiabhi Okhale, Salawu Oluwakanyinsola Adeola

**Affiliations:** 1 *Department of Pharmacology and Toxicology, National Institute for Pharmaceutical Research and Development, P.M.B. 21, Garki -Abuja, Nigeria*; 2 *Department of Medicinal Plant Research and Traditional Medicine, National Institute for Pharmaceutical Research and Development, P.M.B. 21, Garki -Abuja, Nigeria*

**Keywords:** *Anxiety*, *Locomotion*, *Memory*, *Parkia biglobossa*, *Spontaneous alternation*, *behaviour*, *Y-maze*

## Abstract

**Objective**: *Parkia biglobossa* stem bark decoction is a popular medicinal plant preparation used as calming agent for tensed patients in traditional medicine. The aim of this study was to evaluate the effects of aqueous stem bark extract of Parkia biglobossa (AEPB) and its active fraction AEPBF3 on anxiety, spontaneous alternation behavior, and locomotor activity. The open field apparatus was used to evaluate effects of AEPB and AEPBF3 on locomotion. The APBE and the active fraction AEPBF3 were standardized using reverse phase high performance liquid chromatography to establish finger print to ascertain identity and stability of the extracts over time.

**Materials and Methods:** The oral median lethal doses (LD_50_) of AEPB and AEPBF3 were evaluated using modified Lorke’s method in rats. The effect of APBE (50-200 mg/kg p.o.), APBEF3 (25 and 50 mg/kg p.o.), diazepam (2.5 mg/kg, i.p.), and 10 ml normal saline/kg on anxiety-like behavior, spontaneous alternation behavior, and locomotion activity were evaluated in rats on elevated plus maze (EPM), Zero-maze, Y-maze, and open field apparatus, respectively. The oral LD_50_ values of AEPB and AEPBF3 were estimated to be 5000 mg/kg and 3800 mg/kg body weight in rats, respectively.

**Results**: AEPB and AEPBF3 significantly (F6, 41=2342, p<0.0001) increased time spent in the open arm of EPM and significantly (F6, 41=2323, p<0.0001) increased time spent in open arms of the Zero maze. The AEPB and AEPBF3 administration produced significant increase (F5, 35=154, p<0.0001) in spontaneous alternation behavior in rats. The AEPB extract and its fraction AEPBF3 significantly increased total locomotor activity (F6, 41=413, p<0.0001) and rearing (F6, 41=150, p<0.0001) in the open field apparatus.

**Conclusion: **The results of the present study provided evidence for anxiolytic and nootropic effects of the AEPB and AEPBF3, thus providing scientific basis for its continuous use in the management of neuropsychiatric disorders characterized by apprehension and amnesia.

## Introduction

Anxiety is a feeling of apprehension, uncertainty, and fear characterized by physical symptoms such as palpitations, sweating, and feelings of stress (Sivaraman et al., 2012 ▶). Anxiety disorder is considered along with other mental diseases as leading global healthcare burden (Tijani et al., 2012 ▶; Kimiskidis et al., 2007 ▶). Approximately 450 million people suffer from a mental or behavioral disorder (WHO, 2001). In spite of significant progress in neuropsychopharmacology, only a small portion of the large population suffering from these disorders receives even the most basic treatment (Tijani et al., 2012 ▶). 

Available conventional drugs for the treatment of mental and behavioral disorders possess serious adverse effects such as ataxia, confusion, neuroleptic malignant syndrome, tardive dyskenisia, and impaired cognition (Nain et al., 2011 ▶; Di Pietro and Seaman, 2007 ▶). Associated toxicity with the use of these drugs and treatment cost (Nain et al., 2011 ▶) have made the search for safe, effective, and affordable alternative herbal based agent for anxiety management and future anxiolytic development imperative. Plants that have been evaluated for their potential anti-anxiety activity include *Piliostigma thoningii* (Madara et al., 2013 ▶) and *Crinum zeylanicum* (Tijani et al., 2012 ▶). 

The African locust bean tree, *Parkia biglobosa,* a perennial leguminous tree belonging to the family mimosoideae grows in the savannah region of West Africa (Campbell, 1980). The seed is commonly called kaluwa and the fruit, dorowaor dozim, around the northern region of Nigeria (Ihegwuagu et al., 2009 ▶). The stem bark infusion of P. biglobosa is used as a mouthwash, vapour inhalant for toothache, or for ear complaints. It is macerated in baths for leprosy and used for bronchitis, pneumonia, skin infections, sores, ulcers, bilharzia, washes for fever, and malaria (Tijani et al., 2009 ▶). The husks and pods are good feed for livestock (Fetuga et al.,1974 ▶, Ajaiyeoba et al., 2002 ▶) and the floury pulp can be made into a refreshing drink, rich in vitamin C and sugar (Campbell, 1980). 

*Parkia biglobosa* has been claimed by traditional medicine practitioners to possess excellent calming activity on tensed patients (Orwa et al., 2009 ▶). Previous studies in our laboratories have shown that aqueous stem bark extract of *Parkia biglobossa* possesses antidiarrheal and antimicrobial effects (Tijani et al., 2009 ▶). The objectives of the present study were to evaluate the effects of Parkia biglobossa aqueous stem bark extract and fractions on anxiety-like behavior and short term memory in rats.

## Materials and Methods

**Animals**

Male Wistar rats aged 8-10 weeks weighing (150–200 g) were obtained from the Animal Facility Centre, (NIPRD), Idu, Abuja. The rats were fed standard laboratory diet, given water ad libitum and maintained under laboratory conditions of temperature 23±2 ^o^C, relative humidity 60%, and 12-hour light; 12-hour dark cycle. Food was withheld for 24 h prior to each experiment. All animal experiments complied with the “Principles of Laboratory Animal Care” (NIH Publication No. 85-23, revised in 1996).

**Chemicals**

Diazepam (Sigma, MO, St Louis, USA), normal saline (0.9% sodium chloride), 0.2% v/v formic acid, acetonitrile, methanol, and distilled water were used in the current study.

**Plant material**

The stem bark of *P. biglobosa* was collected from Suleja (Niger State, Nigeria) by Mallam Muazzam Ibrahim, an ethnobotanist in the Department of Medicinal Plant Research and Traditional Medicine, National Institute for Pharmaceutical Research and Development (NIPRD), Idu Industrial Area, Abuja, Nigeria. The leaves, fruits, and stem bark were identified and authenticated by Mrs. Grace Ugbabe, a taxonomist in the same department .Voucher specimen (No. NIPRD/H/6225) was deposited in the central herbarium of NIPRD. The stem bark was cleaned, air-dried at room temperature (22-25 ^o^C) away from sunlight, and pounded into fine powder using mortar and pestle. The powder was stored in an air-tight container and kept at 25^o^C for subsequent use. 

**Extraction method**

Two liters of boiling distilled water was added to 200 g of the sample in a 5-litre flat-bottom flask, stirred with a glass rod, covered, shaken continuously for 6 h using GFL shaker (TUV Product Service, Germany), and then allowed to stand for another 18 h at room temperature. The extract was then filtered using Whatmann filter paper No. 91 (18.5 cm). The filtrate was evaporated to dryness on a water bath to obtain aqueous extract of *Parkia biglobosa* (AEPB) with yield of 9.0% (w/w).

**Phytochemical screening**

Phytochemical screening was carried out on AEPB and AEPBF3 using standard methods (Sofowora, 2008 ▶; Trease and Evans, 1996) for detecting the presence of secondary metabolites: alkaloids, carbohydrates, free reducing sugars, combined reducing sugars, tannins, saponins, glycosides, sterols, terpenes, and flavonoids.

**Fractionation**

Aqueous extract of *Parkia biglobossa* (AEPB) was subjected to column chromatography using the method described by Harborne (1998) ▶. This consisted of Octadecylsilyl (ODS) silica gel stationary phase 300 g and 12.37 g of AEPB. Gradient elution under gravity was performed with 500 ml of each mobile phase mixture in series. 

The mobile phase consisted of distilled water and methanol, starting with distilled water (100%) and 10% increments in methanol. The final elution was performed with 100% methanol. A total of 35 fractions were obtained. The eluates were monitored with thin layer chromatography; eluates with similar TLC profiles were pooled to obtain four fractions (AEPBP F1 to AEPBP F4). However, the yield of the AEPB F4 was insignificant hence was not used in the study. 

**High performance liquid chromatography analysis**

The chromatographic system was Shimadzu HPLC system consisting of Ultra-Fast LC-20AB prominence equipped with SIL-20AC auto-sampler; DGU-20A3 degasser; SPD-M20A UV-diode array detector; column oven CTO-20AC, system controller CBM-20Alite, and Windows LCsolution software (Shimadzu Corporation, Kyoto Japan); column, VP-ODS 5 µm, and dimensions (150×4.6 mm). 

The chromatographic conditions included mobile phase: solvent A: 0.2% v/v formic acid; solvent B: acetonitrile; mode: isocratic; flow rate 0.6 ml/min; injection volume 25 µl of 300 µg/ml solution of AEPB and fractions in warm water; detection UV 254 nm. The HPLC operating condition was at column oven temperature of 40^o^C. The total run time was 15 minutes.

**Behavioural tests**

All the behavioural procedures were carried out between 8:00 am and 12:00 pm in a temperature controlled room (23±1 ^◦^C). The mice were grouped such that each group consisted of equal number of males and females which were separately housed.

**Acute toxicity (LD**_50_ **) study**

Acute toxicity study was carried out according to method described by Lorke (1983) ▶. The study was carried out in two phases. In the first phase, nine rats were randomized into three groups (three rats per group) and were given 10, 100, and 1000 mg AEPB /kg body weight orally (via cannula), respectively. Animals were observed for 24 h after treatment for signs of toxicity and mortality. Absence of mortality in animals used for the first phase of the study at 24 h, informed the choice of doses for the second phase, in which 1600, 2900, and 5000 mg AEPB/kg were given orally to another fresh set of three rats per group. 

The rats were also observed for signs of toxicity and mortality. The final median lethal dose (LD_50_) value was calculated as the square root of the product of the lowest lethal dose and the highest non-lethal dose, i.e., the geometric mean of the consecutive doses for which 0 and 100% survival rates were recorded in the second stage. The same procedure was repeated for the AEPBF3 in another set of rats in order to obtain information on its acute toxicity profile and establish its oral median lethal dose (LD_50_).

**Elevated plus maze**

The elevated plus maze is an anxiety paradigm based on the rodent’s natural aversion to a novel and potentially dangerous environment represented by the open and elevated spaces (Lister, 1987 ▶). The elevated plus maze apparatus is a plus (+) shaped wooden structure, consisting of two open arms (40×5×10 cm^3^) and two enclosed arms (40×5×10 cm^3^) extended from a central platform (10×10 cm^2^). 

The maze was elevated 50 cm from the room floor. Rats were habituated to the testing room under dim light for at least 1 h before the test and then randomly divided into seven groups. The rats that served as control group received 10 ml normal saline/kg body weight orally, while the treated rats received AEPB (50, 100, and 200 mg/kg body weight orally) and AEPB F3 (25, 50 mg/kg), and diazepam (2.5 mg/kg body weight i.p.). One hour after oral treatment with AEPB and AEPBF3 and thirty minutes after intraperitoneal administration of diazepam, each rat was placed at the center of the maze, facing one of the open arms and allowed to explore the maze freely for a 5-min testing period. The time spent in open and enclosed arms were recorded. The maze was thoroughly cleaned between tests with a tissue paper moistened with 70% ethanol.

**Elevated zero maze**

This study was carried out according to the method described by Singh et al., (2007). Rats were randomly divided into eleven groups of six rats each. One hour before this test, rats were treated with graded doses of AEPB (50, 100, and 200 mg/kg, orally) and AEPB F3 (25 and 50 mg/kg orally). 

The control group received 10 ml normal saline/kg while standard reference drug diazepam (2.5 mg/kg, i.p.) was administered thirty minutes before the test. Elevated zero maze is a modification of the elevated plus maze model of anxiety in rodents. The novel design consists of an elevated (50 cm above the floor) circular platform (6 cm width and 40 cm inner diameter) that is equally divided into four quadrants. Two quadrants on opposite sides of the platform are enclosed by 12 cm high walls while the other two quadrants are opened and bordered by 0.6 cm high lip. Thus removing any ambiguity in the interpretation of the time spent in the central square of the traditional design (elevated plus maze) and allowing uninterrupted exploration. One hour after drug administration, each rat was placed at the center of the open arm (facing toward the closed chamber). 

The times spent in both open and closed arms of the maze were manually recorded. The maze was thoroughly cleaned between tests with a tissue paper moistened with 70% ethanol.

**Open-field test (OF)**

Locomotor activity and exploratory behavior were assessed in an open field by the method described by Souza et al. (2010) ▶. The OF apparatus consist of a clear glass box (45×45 cm^2^). The floor was divided by lines drawn into 9 equally sized squares. Twenty-four rats were randomly divided into four groups of six rats each. One hour before test session, rats were treated orally with graded doses of AEPB (50, 100, and 200 mg/kg) and AEPB F3 (25 and 50 mg/kg) while the control received 10 ml normal saline/kg orally. One hour later each rat was placed individually in the center of the apparatus and observed for 5 min to record the locomotor (number of squares crossed with four paws) and exploratory activities (indicated by frequency of rearing) (Walsh and Cummins, 1976 ▶; Souza et al., 2010 ▶)

**Y-maze task**

The Y-maze test was carried out using the method described by Heo et al. (2009) ▶. Y-maze test is used to measure immediate spatial working memory, which is a short term memory (Sarter et al., 1988). The Y-maze is a three-arm horizontal maze (40 cm long and 5 cm wide with walls 10 cm high) in which the three arms are symmetrically separated at 120^0^. Rats were initially placed within one arm (A), and the arm entry sequence (e.g., ABC CAB, where letters indicate arm codes) and the number of arm entries were recorded manually for each rat over a 6-min period. The maze arms were cleaned with 70% ethanol between tasks to remove residual odors. Alternation was determined from successive entries into the three arms on overlapping triplet sets in which three different arms are entered. An actual alternation was defined as entries into all three arms consecutively (i.e., ABC, CAB, or BCA but not BAB). An entry was defined as placing all four paws within the boundaries of the arm. One hour before this test, rats were treated with graded doses of Parkia biglobossa aqueous stem bark extract (50, 100, and 200 mg/kg, orally) and AEPB F3 (25 and 50 mg/kg orally). 

The control group received 10 ml normal saline/kg. The percentage alternation for each rat was determined as the ratio of actual to possible alternations (defined as the total number of arm entries minus 2), multiplied by 100 as shown by the following equation: % Alternation = [(Number of alternations) / (Total arm entries-2)] × 100 (Kim et al., 2007 ▶; Heo et al., 2009 ▶)

**Statistical analysis**

All data were expressed as mean±SEM. Statistical analysis was carried out using one-way analysis of variance (ANOVA). Any significant difference between means was assessed by student’s t-test at 95% level of significance

## Results

**The yield of extracts**

The yields of the extracts were 9.0% w/w, 21.3% w/w, 23.2% w/w, and 38.9% w/w for crude, PF1, PF2, and PF3, respectively. Each of the fractions was subsequently evaluated for anxiolytic-like effect using elevated plus maze in order to select the most active fraction for further studies. Fraction PF3 designated as AEPBF3 was found to be most active against anxiety-like behavior and was eventually selected for further studies. 

**Phytochemical composition**

Results of phytochemical screening of the aqueous extract of *Parkia biglobosa* stem bark (AEPB) and the PF3 are shown in Table 1. As illustrated, the major phytochemical constituents of AEPB are tannins, alkaloids, and saponins. AEPBF3 contains tannins and saponins.

**Table 1:** Results of phytochemical screening of the aqueous extract of Parkia biglobosa stem bark (AEPB) and AEPBF3

Secondary Metabolites	AEPB	AEPB F3
Carbohydrates Free reducing sugars Combined reducing sugars Tannins Alkaloids Saponins Terpenes Sterols Anthraquinones Cardiac glycosides Flavonoids	+ + ++ +++ + + - - - - -	- + ++ +++ - + - - - - -

+ Slightly present, ++ present, +++ highly present, - Absent. AEPBF3 exhibited the highest anxiolytic-like effect in rats on EPM of all the column chromatographic fractions of AEPB

**HPLC analysis**

**Figure 1:**
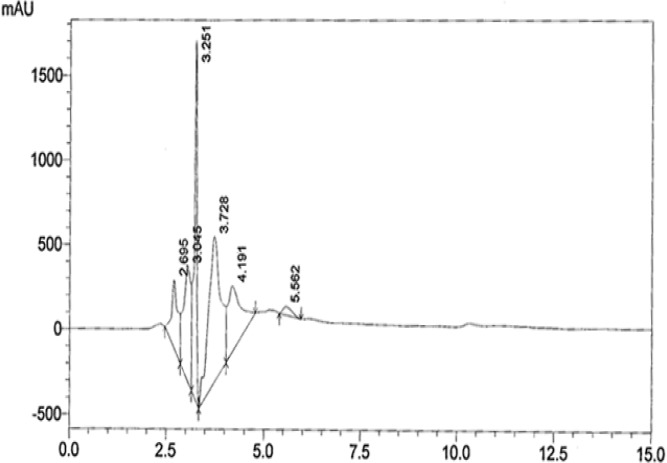
HPLC chromatogram of AEPB

**Figure 2:**
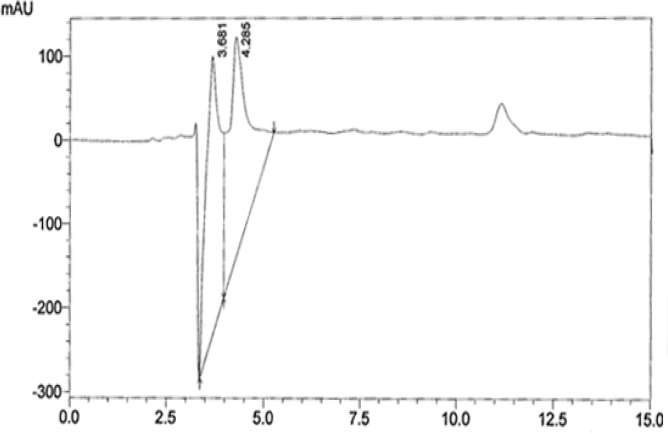
HPLC chromatogram of AEPBF3

According to the HPLC spectrum of AEPB six peaks were detected as shown (Figure 1) with retention times of 2.96, 3.04, 3.25, 3.72, 4.19, and 5.56 minutes. The HPLC spectrum of AEPB F3 showed two major compounds as two peaks with retention times of 3.68 and 4.29 minutes (Figure 2) corresponding to 3.72 and 4.19 of AEPB. 

**Acute toxicity study**

The AEPB extract did not produce any sign of toxicity at the doses administered orally. No mortality was also recorded. The oral median lethal dose (LD_50_) of AEPB was estimated to be greater than 5000 mg/kg body weight in rats. The fraction AEPB F3 at 5000 mg/kg produced exaggerated excitement followed by calmness, immobility, comatose, convulsion, and death. The oral median lethal dose (LD50) of AEPBF3 was determined to be 3800 mg/kg body weight. 

**Effect of AEPB and AEPBF3 on elevated plus maze (EPM)**

**Figure 3:**
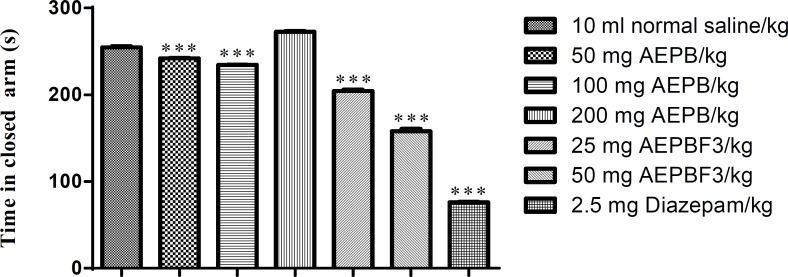
Effect of AEPB and AEPB F3 on time spent in closed arm of the EPM

The AEPB extract as well as its fraction AEPBF3 significantly (F6, 41=2342, p<0.0001) decreased the time spent in the closed arm of the elevated plus maze (EPM). However, 200 mg/kg of AEPB significantly increased the time spent in the closed arm of the EPM. Diazepam was more potent in reducing the time spent in closed arm of the EPM than both the AEPB and AEPB F3 (Figure 3).

**Figure 4:**
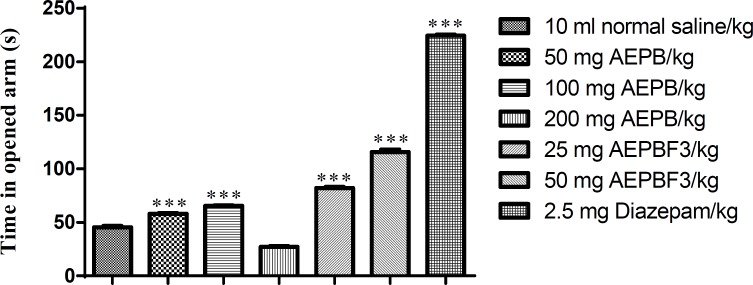
Effect of AEPB and AEPBF3 on time spent in opened arm of the EPM.

AEPB, and AEPB F3 significantly (F6, 41=2331, p<0.0001) increased the time spent in the closed arm of the elevated plus maze (Figure 4).

**Effect of AEPB and AEPB F3 on elevated zero maze**

**Figure 5:**
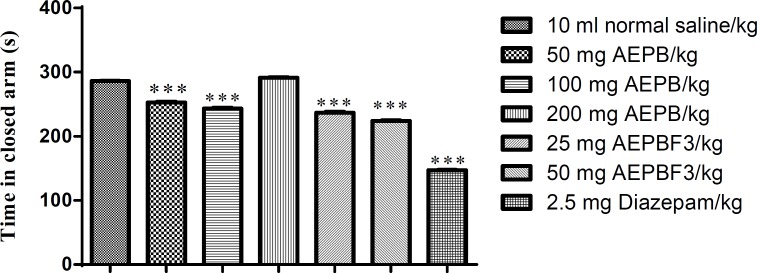
Effect of AEPB and AEPBF3 on time spent in closed arm of zero maze

AEPB and AEPBF3 significantly (F6, 41=2323, p<0.0001) decreased the time spent in the closed arm of the elevated zero maze except for 200 mg AEPB/kg where significant increase in time spent in the open arm was observed (Figure 5).

*** Significantly different from the control at p<0.0001.

*** Significantly different from the control at p<0.0001.

*** Significantly different from the control at p<0.0001.

**Figure 6:**
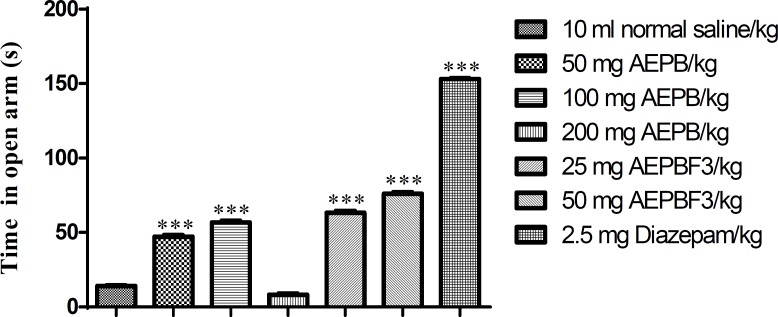
Effect of AEPB and AEPBF3 on time spent in open arm of zero maze

AEPB and AEPBF3 significantly (F6, 41=2323, p<0.0001) increased the time spent in the open arm of the elevated zero maze except for 200 mg AEPB/kg which decrease time spent in the open arm of elevated zero maze significantly, while diazepam increased time spent on open arm of the maze (Figure 6).

**Effect of AEPB and AEPBF3 on total locomotive activity**

**Figure 7:**
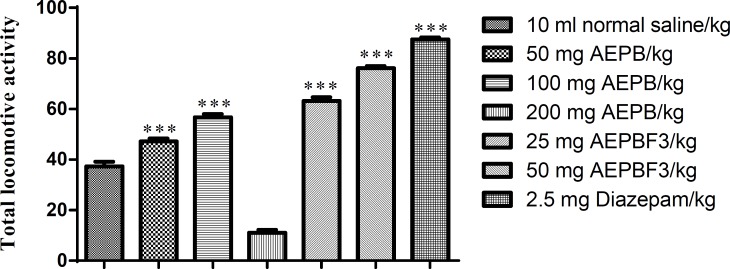
Effect of AEPB and AEPBF3 on total locomotive activity on open field apparatus

The AEPB extract at 50 and 100 mg/kg and its fraction AEPBF3 at 25 and 50 mg/kg significantly (F6, 41=413, p<0.0001) increased the total locomotive activity of rats on open field apparatus (Figure 7).

**Effect of AEPB and AEPBF3 on rearing**

**Figure 8:**
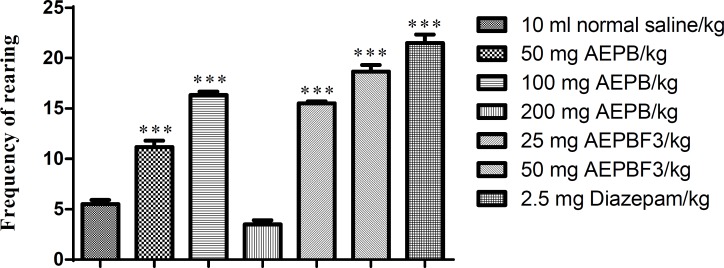
Effect of AEPB and AEPBF3 on rearing

AEPB and AEPBF3 significantly (F6, 41 =150, p<0.0001) increased frequency of rearing (Figure 8).

**Effect of Parkia biglobossa on spontaneous alternation behavior of rat in Y-maze**

**Figure 9:**
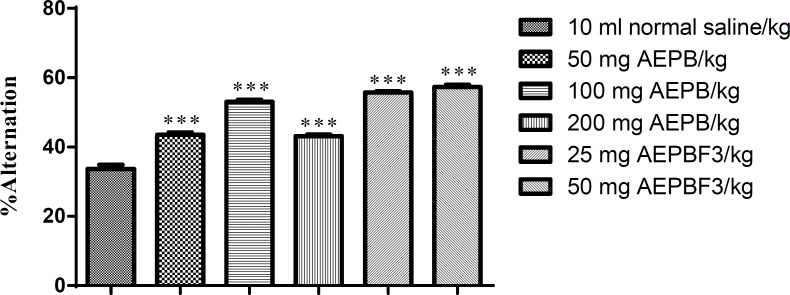
Effect of AEPB and AEPBF3 on spontaneous alternation behavior (SAB). ***Significantly different from the control at p<0.0001.

The AEPB (50, 100, and 200 mg/kg) and AEPBF3 (25 and 50 mg/kg) significantly (F5, 35=154, p<0.0001) increased the spontaneous alternation behavior in rats compared with the control (Figure 9).

***Significantly different from the control at p<0.0001.

***Significantly different from the control at p<0.0001.

*** Significantly different from the control at p<0.0001.

## Discussion

The plant material was extracted with hot water in a similar manner which traditional healers prepares and administers it. The decoction obtained was however freeze-dried to preserve thermo-labile components of the extract and avoid fermentation and hydrolysis. The obtained dried extract was then subjected to high performance liquid chromatography (HPLC). The chromatogram of the aqueous extract revealed an identification parameter for finger printing. This is fundamental for quality assurance in terms of further collection to avoid substitution and track changes due to variations in storage condition. The finger print may also be used to establish stability of the extract over a period of time as earlier shown by Tijani et al. (2013) ▶. 

The phytochemical screening of the crude extract and the active fraction revealed the presence of carbohydrates, free reducing sugars, combined reducing sugars, tannins, alkaloids, and saponins. The therapeutic benefits of medicinal plant extracts used as traditional remedies have been attributed to a combination of active secondary metabolites (Amos et al., 2001 ▶). The saponins have been shown to possess sedative properties, antagonistic effects against amphetamine, and decreased spontaneous motor activity in experimental animals (Wagner et al., 1985). Moreover, flavonoids, tannins, alkaloids, sterols, terpenes, and resins in medicinal plants were shown by Duke. (1992) ▶ to possess significant anti-nociceptive, sedative, and anti-psychotic effects. The results of the behavioral studies revealed that the aqueous stem bark extract of Parkia biglobossa produced significant anxiolytic effect in rats on elevated plus maze, elevated zero- maze, and the open field as well as improved spatial memory of rats on Y-maze. 

The models of anxiety used in this study are widely used to screen the new anxiolytic drugs. These models are quite sensitive and relatively specific to all major classes of anxiolytic drugs and are therefore appropriate for the studies. The oral median lethal dose (LD_50_) of the extract in rats was estimated to be greater than 5000 mg/kg, indicating that the extract is practically non-toxic acutely (Matsumura, 1975 ▶; Corbett et al., 1984 ▶). This wide margin of safety may have been responsible for its continuous wide spread use in folk medicine for the management of neuropsychiatric disorders.

*Parkia biglobossa* aqueous stem bark extract at 50 and 100 mg/kg significantly increased the time spent in the open arm of the elevated plus maze. The extract however produced a significant increase in time spent in the enclosed arm of the maze at 200 mg/kg. This observation is consistent with standard anxiolytics behaving similar to benzodiazepines with anxiolytic effect at low doses and anxiogenic or sedative effect at higher doses (Madara et al., 2013 ▶, Tijani et al., 2012 ▶). 

The partial purification of the extract through column chromatography and eventual evaluation of the fractions obtained yielded a highly potent fraction designated as aqueous extract of *Parkia biglobossa* fraction 3 (AEPBF3). The AEPBF3 at doses of 25 and 50 mg/kg produced anxiolytic effects characterized by increased time spent in the open arms of the elevated plus maze. The anxiolytic activities of AEPB and AEPBF3 were less than that of diazepam. The recorded lower activities may be due to the presence of phytochemicals with diverse pharmacological effects in AEPB and AEPBF3 on treated mice (Tijani et al., 2008 ▶).

The elevated plus maze (EPM) test represents one of the most widely used animal models for screening anxiolytics (Lister, 1987 ▶). This test is able to reproduce anxiolytic or anxiogenic effects in rodents such that anxiolytics produce increase the time spent in the open arm of the elevated plus maze, while anxiogenic substances produce the opposite effect (Lister, 1987 ▶, 1990; Pellow and File, 1986 ▶). 

The indices of anxiety (percentage of open-arm entries, and percentage of time spent in the open arm) are sensitive to agents and are thought to act via the GABAA receptor complex, justifying the use of diazepam (DZP) as a positive control in this study. Diazepam, a benzodiazepine binds to GABAA receptors to increase the frequency of chloride channel openings resulting in hyperpolarization. It increased the frequency of open-arm entries and the time spent in the open arms (Crawley & Goodwin, 1980 ▶), confirming its anxiolytic effects. The *Parkia biglobossa* aqueous stem bark extract had similar effects on these parameters.

The anxiolytic effect of the extract and AEPBF3 was further confirmed by the results obtained from the use of the elevated zero maze. The zero-maze has two advantages over the elevated plus maze: no ambiguity associated with the interpretation of the time spent in the central square of the elevated plus maze and allowance of uninterrupted exploration. The extract at 50 and 100 mg/kg produced anxiolytic-like effect which is clearly defined by the increased time spent in the open quadrant of the zero -maze. At 200 mg/kg, the extract exerted anxiogenic –like effect in the various test methods used in the study. This observation may be due to the extreme spectrum of anxiolytic sedative effects characterized by sedation-like behavior. This is consistent with the effect of sedative-anxiolytics.

The open-field apparatus provides information on anxiety-related behaviour characterized by natural aversion of rodents to an open brightly lit area (Asano, 1986; Choleris et al., 2001 ▶). Animals are thus afraid of the centre and spend more time in the protective corners and in freezing state. Anxiolytics increase total locomotive activity resulting in a reduction of time spent in corners, an increased time spent in the center and a decreased time spent in freezing state. The extract at 50 and 100 mg/kg as well as the partially purified AEPBF3 at 25 and 50 mg/kg body weight increased total locomotive activity and increased rearing of treated rats. This observation further confirmed the anxiolytic potential of AEPB and AEPBF3. Natural products of plant origin may elicit anxiolytic effects via interaction with some endogenous mediators such as GABAergic and serotonergic pathways in the body (Tijani et al., 2012 ▶; Kadaba 1994 ▶). Most anxiolytics enhances response to GABA through facilitation of the opening of GABA activated chloride ion channels. Thus, the present study showed that AEPB and AEPBF3 possessed potent anxiolytic effects as evidenced in the results obtained from the various used models.

Spontaneous alternation behavior is regarded as a measure of short-term memory in rodents (Heo et al., 2009 ▶; Hritcu et al., 2007 ▶). A rat must remember the least recently visited arm in order to alternate the arm choice (Hooper et al., 1996 ▶; Lee et al., 2010 ▶). AEPB and AEPBF3 administered to healthy rats produce significant increase in spontaneous alternation behavior. Thus, the tannins and saponins present in AEPB and AEPBF3 may be responsible for the observed anxiolytic-like effects in treated rats. This observation is in line with early report by Ambavade et al. (2008) ▶ on the anxiolytic effects of tannins. Similarly, Amos et al. (2001), Cha et al. (2004) ▶, and Churchill et al. (2002) ▶ have associated anxiolytic and nootropic activities of plant extracts to the presence of saponins.

It may therefore be concluded that the aqueous stem bark extract of *Parkia biglobossa* (AEPB) as well as its partially purified fraction AEPBF3 possess anxiolytic and nootropic effects which can be attributed to the presence of tannins and saponins thus providing rational scientific evidence for its continuous use in folkloric medicine for calming tensed individuals. 

## Conflict of interest

The authors alone are responsible for the content of this manuscript and have declared no conflict of interest.

## References

[B1] Ajaiyeoba Edith (2002). Phytochemical and antibacterial properties of Parkia biglobosa and Parkia bicolor leaf extracts. Afr J of Biomed Res.

[B2] Ambavade D Shirikumar, Mhetre A Nilesh, Muthal P Amol, Bodhankar L Subhash (2008). Anxiolytic activity of root extracts of Decalepsi hamiltonii W.A. in mice. Pharm online.

[B3] Amos S, Adzu B, Binda L, Wambebe C, Gammaniel K (2001). Neuropharmacological effect of aqueous extract of Spharenthus senegalensis in mice. J Ethnopharmacol.

[B4] Cha HY, Seo JJ, Park JH, Choi KJ, Hong JT, Oh JK (2004). Anxiolytic effects of total Saponin fraction from Ginseng Radix Rubra on the elevated plus –maze model in mice. Ginseng Res.

[B5] Choleris E, Thomas AW, Kavaliers M, Prato FS (2001). A detailed ethological analysis of the mouse open field test: Effects of diazepam, chlordiazepoxide and an extremely low frequency pulsed magnetic field. Neurosci Behav Rev.

[B6] Churchill JD, Gerson JL, Hinton KA, Mifek JL, Walter MJ, Winslow CL, Deyo RA (2002). The nootropic properties of ginseng saponin Rb 1 are linked to effects on anxiety. Integr Physiol Behav Sci.

[B7] Corbett JR, Wright, Baille AC (1984). The Biochemical mode of action of pesticides.

[B8] Crawley JN (1999). Behavioral phenotyping of transgenic and knockout mice: Experimental design and evaluation of general health, sensory functions, motor abilities and specific behavioural tests. Brain Res.

[B9] Di Pietro C, Seamans JK (2007). Dopamine and serotonin interactions in the prefrontal cortex: Insights on antipsychotic drugs and their mechanism of action. Pharmacopsych.

[B10] Duke JA (1985). Handbook of Biologically active Phytochemicals and Their Activities.

[B11] Fetuga BL, Babatunde GM, Oyenuga VA (1974). Protein quality of some unusual protein foods -African Locust bean seed. Brit J Nutri.

[B12] Harborne JB (1998). Phytochemical Methods, A Guide to Modern Technique of Plant Analysis.

[B13] Heo H, Shin Y, Cho W, Choi Y, Kim H, Kwon YK (2009). Memory improvement in ibotenic acid -induced model rats by extracts of Scutellariabaicalensis. J Ethnopharmacol.

[B14] Hooper N, Fraser C, Stone T (1996). Effects of purine analogues on spontaneous alternation in mice. Psychopharmacology.

[B15] Hritcu L, Clicinschi M, Nabeshima T (2007). Brain serotonin depletion impairs short-term memory, but not long-term memory in rats. Phys Behav.

[B16] Kadaba, BKA (1994). A safe herbal treatment for anxiety. Brit J Phytother.

[B17] Kim DH, Hung TM, Bae KH, Jung JW, Lee S, Yoon BH, Cheong JH, Ko KH, Ryu JH (2006). Gomisin A improves scopolamine-induced memory impairment in mice. Eur J Pharmacol.

[B18] Kimiskidis VK, Triantafyllou NI, Kararizou E, Gatzonis S, Fountoulakis KN, Siatouni A, Loucaidis P, Pseftogianni D, Vlaikidis N, Kaprinis GS (2007). Depression and anxiety in epilepsy: the association with demographic and seizure-related variables. Annals Gen Psychiatry.

[B19] Lanza M, Regli P, Busson F (1962). Hydroxyproline content in the protein of the fruit pulp of Parkia biglobosa. Med. Trop.

[B20] Lee M, Yun B, Zhang D, Liu L, Wang Z, Wang C, Gu L, Wang C, Mo E, Ly S, Sung C (2010). Effect of aqueous antler extract on scopolamine-induced memory impairment in mice and antioxidant activities. Food Sci Biotechnol.

[B21] Lister RG ( 1987). The use of a plus-maze to measure anxiety in the mouse. Psychopharmacol (Berl).

[B22] Lorke D (1953). A New Approach to Practical Acute Toxicity Testing. Arch Toxicol.

[B23] Madara AA, Tijani AY, Bitrus A, Salawu OA (2013). Pharmacological effects of Piliostigma thoningii leaf extract on anxiety-like behaviour and spatial memory in Wistar albino rats. Phytopharmacology.

[B24] Matsumura F (1975). Toxicology of Insecticides.

[B25] Nain P, Kumar S, Nain J, Kumar S (2011). Evaluation and comparison of Anxiolytic effect of flax seed oil and perilla oil in rats. Inter Res J Pharmacy.

[B26] Ihegwuagu NE, Moses OO, Martins OE, Olobayo OK (2009). Isolation and evaluation of some physicochemical properties of Parkia biglobosa starch. Pure Appl Chem.

[B27] Orwa C, Mutua A, Kindt R, Jamnadass R, Simons A (2009). Agroforestree Database: a tree reference and selection guide version 4.0. http://www.worldagroforestry.org/af/treedb/.

[B28] Pellow S, File SE (1986). Anxiolytic and anxiogenic drug effects on exploratory activity in an elevated plus maze: a novel test of anxiety in rats. Pharmacol Biochem Behav.

[B29] Silvaraman D, Muralidharan P, Habibur R (2012). Evaluation of the anxiolytic effect of methanolic leaf extract of Ficus hispida Linn in corticosterone induced anxiety in young adult rats. Pharmacologia.

[B30] Kulkarni SK, Singh K, Bishnoi M (2007). Elevated Zero maze: A paradigm to evaluate anti-anxiety effects of drugs. Methods Find Exp Clin Pharmacol.

[B31] Sofowora A (2008). Medicinal Plants and Traditional Medicine in Africa.

[B32] Souza G, Christina A, Cesar AB, Marlon RL, Gilson Z, Christina WN (2010). Diphenyl diselenide improves scopolamine-induced memory impairment in mice. Behav Pharmac.

[B33] Tijani AY, Okhale SE, Oga FE, Tags SZ, Salawu OA, Chindo BA (2008). Anti-emetic Activity of Grewia lasiodiscus Root Extract and Fractions. Afr J Biotech.

[B34] Tijani AY, Okhale SE, Salawu TA, Onigbanjo HO, Obianodo LA, Akingbasote JA, Salawu OA, Okogun JI, Kunle FO, Emeje M (2009). Antidiarrhoeal and antibacterial properties of crude aqueous stem bark extract and fractions of Parkia biglobosa (Jacq.) R.Br.Ex G. Don. Afr J of Pharm and Pharmacol.

[B35] Tijani AY, Salawu OA, Anuka AJ, Isah MH (2012). Sedative and Anxiolytic effects of Crinum zeylanicum. Med Chem Drug Discov.

[B36] Walsh RN, Cummins RA (1976). The open-field test: a critical review. Psychol Bull.

[B37] World Health Report (2001). Mental health: New understanding new hope.

